# Stable cell fate changes in marrow cells induced by lung-derived microvesicles

**DOI:** 10.3402/jev.v1i0.18163

**Published:** 2012-04-16

**Authors:** Jason M. Aliotta, Mandy Pereira, Ming Li, Ashley Amaral, Arina Sorokina, Mark S. Dooner, Edmund H. Sears, Kate Brilliant, Bharat Ramratnam, Douglas C. Hixson, Peter J. Quesenberry

**Affiliations:** 1 Division of Hematology and Oncology, Rhode Island Hospital, The Warren Alpert Medical School of Brown University, Providence RI 02903, USA; 2 Division of Pulmonary, Sleep and Critical Care Medicine, Rhode Island Hospital, The Warren Alpert Medical School of Brown University, Providence RI 02903, USA; 3 Laboratory of Retrovirology, Division of Infectious Diseases, The Warren Alpert Medical School of Brown University, Providence RI 02903, USA

**Keywords:** bone marrow cells, lung, microvesicles, bone marrow transplant, transcription factor

## Abstract

**Background:**

Interest has been generated in the capacity of cellular-derived microvesicles to alter the fate of different target cells. Lung, liver, heart and brain-derived vesicles can alter the genetic phenotype of murine marrow cells; however, the stability of such changes and the mechanism of these changes remain unclear. In the present work, we show that lung-derived microvesicles (LDMV) alter the transcriptome and proteome of target marrow cells initially by mRNA and regulator(s) of transcription transfer, but that long term phenotype change is due solely to transfer of a transcriptional regulator with target cell.

**Methods/results:**

In vivo studies: Whole bone marrow cells (WBM) were co-cultured with LDMV (both isolated from male C57BL/6 mice) or cultured alone (control). One week later, cultured WBM was transplanted into lethally-irradiated female C57BL/6 mice. Recipient mice were sacrificed 6 weeks later and WBM, spleens and livers were examined for the presence of lung-specific gene expression, including surfactants A, B, C and D, aquaporin-5, and clara cell specific protein, via real-time RT-PCR. Immunohistochemistry was also performed on lungs to determine the number of transplanted marrow-derived (Y chromosome+) type II pneumocytes (prosurfactant C+). Mice transplanted with LDMV co-cultured WBM expressed pulmonary epithelial cell genes in the cells of their bone marrow, livers and spleens and over fivefold more transplanted marrow-derived Y+/prosurfactant C+cells could be found in their lungs (vs. control mice). In vitro studies: WBM (from mice or rats) was cultured with or without LDMV (from mice or rats) for 1 week then washed and cultured alone. WBM was harvested at 2-week intervals for real-time RT-PCR analysis, using species-specific surfactant primers, and for Western Blot analysis. Proteomic and microRNA microarray analyses were also performed on cells. LDMV co-cultured WBM maintained expression of pulmonary epithelial cell genes and proteins for up to 12 weeks in culture. Surfactant produced at later time points was specific only to the species of the marrow cell in culture indicating de novo mRNA transcription. These findings, in addition to the altered protein and microRNA profiles of LDMV co-cultured WBM, support a stable transcriptional mechanism for these changes.

**Conclusions:**

These data indicate that microvesicle alteration of cell fate is robust and long-term and represents an important new aspect of cellular biology.

Over the past decade, many investigators have reported that in rodent models, transplanted bone marrow cells are capable contributing to the cellular component of the injured lung (1–3). These and other studies of adult stem cell “plasticity” are considered controversial, in part due to the lack of a known mechanism explaining the ability of marrow cells to assume phenotypic characteristics of non-hematopoietic cells. Our group has demonstrated that murine marrow cells that are co-cultured with murine lung cells express pulmonary epithelial cell-specific mRNA and protein (4). Microvesicles shed from lung cells in culture are responsible for these changes as only cells that have internalized microvesicles demonstrate these changes (5). These studies add to a growing body of literature which highlights the importance of microvesicles as mediators of cellular communication and entities that are capable of altering the phenotype of cells with which they interact.

Microvesicles are membrane-enclosed packets of cellular information which are capable of altering cell fate or phenotype (6). Microvesicles originating from platelets and red blood cells were initially felt to represent “cellular junk” but their biological relevance is beginning to be recognized (7, 8). They are now known to originate from many different cells and do so under the influence of a variety of exogenous stressors including hypoxia, shear stress, irradiation, chemotherapeutic agents and cytokines (6). Many different subpopulations of cellular-derived vesicles other than microvesicles have been described including exosomes, ectosomes, membrane particles and apoptotic vesicles (9, 10). They have been characterized by size, density, morphology by electron microscopy, density in sucrose, sedimentation with ultracentrifugation, lipid composition, protein markers and intracellular origins. Exosomes are 30–100 nm in diameter and are derived from endocytic vesicles. They are released upon fusion of multivesicular bodies with plasma membranes. Microvesicles are 100 nm to 1 µm in diameter and are released from the cell surface by membrane blebbing in a calcium flux and calpain-dependent manner. Expression of floppase, flippase and scramblase, 3 cell membrane-based phopholipidic pumps responsible for maintaining transbilayer lipid distribution, becomes imbalanced. As a result, phophatidylserine is redistributed to the external membrane leaflet leading to membrane blebbing and microvesicle release (11). Since the majority of techniques used to isolate cellular-derived vesicles include differential speeds of ultracentrifugation, most preparations that have been described in the literature are inherently heterogeneous. Our lab's preparations contain vesicles that range from 40 to 440 nm in diameter and appear to include a combination of exosomes and microvesicles. However, we have elected to use the term microvesicle in this paper as the majority of vesicles in our preparations are greater than 100 nm in diameter. The contents of cellular-derived vesicles are a reflection of the cell of origin as they contain elements of cell membrane (including antigens, receptor and lipid rafts) and cytoplasmic components (including secreted proteins mRNA, microRNA, DNA, apoptotic DNA, mitochondrial DNA and protein). Vesicles can act as a vehicle to deliver these elements to other cells where they are capable of inducing changes in cellular phenotype (12). Although vesicles can enter target cells by a variety of different mechanisms, interactions between specific receptors and ligands appear to be crucial (13).

Our group has also found that murine lung-derived microvesicles (LDMV) contain DNA, microRNA and lung-specific mRNA and protein and that the contents of LDMV are passed along to cells which internalize them (5). Although the same mRNA species found in LDMV can also be found in cells which have internalized LDMV, studies using transcriptional blocking agents have demonstrated transcriptionally active agents are likely to be responsible for these changes in cellular phenotype (5). Co-cultures which used tissues from different rodent species and were analyzed with tissue-specific PCR primers supported this notion. In these studies, we found that surfactant mRNA detected in the co-cultured marrow cells was specific to the rodent species of the lung tissue used in co-culture as well as the rodent species of co-cultured marrow cells, indicating that these mRNA species were both transferred via LDMV and produced de novo (5). We have also found that the microRNA profile and proteome of marrow cells 2 days after LDMV internalization are vastly different compared with unmanipulated cells, further suggesting that an LDMV-based transcriptional agent is transferred to cells in culture. As the co-cultures established in these studies were relatively short-lived (all under 7 days), a critical question is whether these changes are transient or persistent. Using a long-term in vitro co-culture and an in vivo transplant model, studies presented in this paper demonstrate that these changes are persistent.

## Methods

### Experimental animals

All mouse studies were approved by the Institutional Animal Care and Use Committee at Rhode Island Hospital (CMTT numbers 0090-10, 0211-08). Six-to-eight-week-old male C57BL/6 mice or male Fischer-344 rats (Jackson Laboratories) were used for all studies. Animals had ad libitum access to food and water and were given 1 week to acclimate prior to experimental treatment or euthanasia. Euthanasia was performed using CO_2_ inhalation or isofluorane inhalation followed by cervical dislocation.

### Tissue collection

For solid organ harvest, after euthanasia, blood was flushed from the vasculature using 10 ml of ice-cold 1×Dulbecco's phosphate-buffered saline (1×PBS, Invitrogen) infused thought the right ventricle. Lungs and livers were collected and placed in ice-cold PBS supplemented with 5% heat-inactivated fetal calf serum (HICFS, Hyclone) and 1% penicillin–streptomycin (PS, Invitrogen). For whole bone marrow cell (WBM) harvest, tibiae, femurs, iliac crests and spines were collected and all surrounding muscle removed with sterile gauze. Bones were placed in ice-cold 1×PBS/5% HIFCS/1% PS and crushed using mortar and pestle. Cells were strained through 40 µm cell strainer (Allegiance) placed over 50 ml conical tube (Fisher) then centrifuged at 300*g* for 10 min at 4°C.

### Lineage depletion

Mononuclear cells were isolated from WBM by discontinuous density centrifugation at 1,000*g* for 30 min at room temperature using OptiPrep (Accurate Chemical). Mononuclear cells were then lineage depleted (Lin–) by adding the following antibodies rat-anti mouse antibodies: anti-Ter119, B220, Mac-1, Gr-1, CD4, and CD8 (BD Biosciences). After 15 min of incubation on ice, Dynabead M450 anti-rat IgG (Dynal) was added and lineage positive cells were removed by a magnetic column. Remaining Lin– cells were counted and percent viability determined was using Trypan Blue stain (Gibco).

### Lung-derived microvesicle (LDMV) isolation

After euthanasia, lungs were filled with dispase (Sigma) though a hole in the trachea using a blunted 18-gague needle attached to a 3 cc syringe. Lungs were then removed and dispase-digested for an additional 45 min on ice. Lungs were then mechanically dissociated with scissors and forceps into a single cell suspension. Cells were passed though a 40 µm cell strainer placed over 50 ml conical tube and washed with PBS by centrifugation at 300*g* for 10 min at 4°C. Lung cells were cultured (1×10^6^ cells/ml) in Bronchial Epithelial Growth Media (BEGM, Lonza), supplemented with 0.5 µg/ml epinephrine, 10 µg/ml transferrin, 5 µg/ml insulin, 0.1 ng/ml retinoic acid, 52 µg/ml bovine pituitary extract, 0.5 µg/ml hydrocortisone, 0.5 pg/ml human recombinant epidermal growth factor and 6.5 ng/ml triiodothyronine, at 37°C/5% CO_2_ for 7 days. Cultured lung cells were then removed by centrifugation at 300*g* for 10 min at 4°C (performed twice) to make LCM. LCM was ultracentrifuged at 10,000*g* for 1 h then at 100,000*g* for 1 h at 4°C in a Thermo Scientific Sorval WX Ultra series ultracentrifuge. The supernatant was discarded and the pellet was resuspended in 1×PBS supplemented with 5 mM HEPES [4-(2-hydroxyethyl) piperazine-1-ethanesulfonic acid, N-(2-Hydroxyethyl) piperazine-N′-(2-ethanesulfonic acid)] (Sigma). The pelleted material (lung-derived microvesicles or LDMV) was ultracentrifuged again at 100,000*g* for 1 h at 4°C, resuspended in DMEM-glutamax (Invitrogen) supplemented with 15% fetal bovine serum (FBS, Hyclone), 1% PS and recombinant murine stem cell factor (SCF, final concentration 50 ng/ml) and used for co-culture.

### In vitro persistence assay

WBM cells (2×10^7^) isolated from male C57BL/6 mice were co-cultured in DMEM-glutamax (Invitrogen) supplemented with 15% FBS, 1% PS and SCF (final concentration, 50 ng/ml) with LDMV isolated from 1 male C57BL/6 murine. Control WBM cells were cultured without LDMV. Cells were incubated at 37°C/5% CO_2_ for 7 days in 6-well culture plates. WBM cells were then removed, washed with 1×PBS by centrifugation at 300*g* for 10 min and placed into secondary culture with the same media, absent LDMV. An aliquot of cells were removed at the onset of secondary culture (0 week time point) and every 2 weeks for a total of 12 weeks. Cells were analyzed by immunohistochemistry, RNA by RT-PCR and protein by western blot.

### Rat/mouse hybrid co-culture

WBM cells (2×10^7^) isolated from male C57BL/6 mice or male Fischer-344 rats were co-cultured in DMEM-glutamax (Invitrogen) supplemented with 15% FBS, 1% PS and SCF (final concentration, 50 ng/ml) with minced lungs or livers isolated from mice or rats (separated by a 400 nm pore size cell-impermeable membrane, Millipore). Control WBM cells from mice or rats were cultured without lungs or livers. Cells were cultured at 37°C/5% CO_2_ for 7 days in 6-well culture plates. WBM cells were then removed, washed with 1×PBS by centrifugation at 300*g* for 10 min and placed into secondary culture with the same media, absent lung or liver. An aliquot of cells were removed at the onset of secondary culture (0 week time point) and every 2 weeks for a total of 12 weeks. WBM cells were analyzed by RT-PCR using mouse or rat-specific primers for the pulmonary epithelial cell-specific genes surfactant B (Sp-B) and C (Sp-C) as well as the liver markers albumin-1 and ATP binding cassette transporter 1 (ABCA1).

### In vivo persistence assay

WBM cells (2×10^7^) isolated from male C57BL/6 mice were co-cultured in DMEM-glutamax (Invitrogen) supplemented with 15% FBS, 1% PS and SCF (final concentration, 50 ng/ml) with LDMV isolated from 1 male C57BL/6 murine lung. Control WBM cells were cultured without LDMV. Cells were cultured at 37°C/5% CO_2_ for 7 days in 6-well culture plates. WBM cells from individual culture wells were then washed with 1×PBS by centrifugation at 300*g* for 10 min at 4°C and counted using Trypan Blue stain. Female C57BL/6 mice were exposed to a 950 cGy of total body irradiation (split doses administered 3 h apart) using a Gammacell 40 Exactor Irradiator at 110 cGy/min (MDS Nordion) and transplanted (via tail vein injection) with 1 culture well of WBM co-cultured with LDMV (n=17) or cultured without LDMV (n=6). In addition, a cohort of irradiated mice (n=6) were transplanted with uncultured WBM cells (control mice). There was no significant difference in the mean cell number (range 4.8×10^6^ to 5.3×10^6^, p≥0.05, wilcoxon) and viability of transplanted cells (range 92–95%, p≥0.05, wilcoxon) in cohorts of recipient mice. Six weeks after transplantation, recipient mice were sacrificed. The pulmonary vasculature of recipient mice was flushed with 1×PBS and lungs were fixed by intratracheal infusion of ice-cold PLP (balanced phosphate solution with 2% paraformaldehyde, sodium m-periodate, and l-lysine) and embedded in paraffin. Bone marrow cells were flushed from both femurs and livers and spleens were removed and placed into PBS supplemented with 5% HICFS. Total RNA was extracted using the RNeasy Mini Kit (Qigen).

### RNA extraction, cDNA amplification and real-time RT-PCR analysis

Extracted RNA was measured for quantity and quality (260/280 ratio) using a Nanodrop ND/1000 spectrophotometer (Thermo scientific). For each sample, 10 ng of RNA was used to amplify cDNA using the High Capacity cDNA transcription kit (Applied Biosystems) in a final volume of 20 µl, per manufacturer's recommendations. Amplification reactions consisted of 1 cycle for 10 min at 25°C, 1 cycle for 120 min at 37°C, and 1 cycle for 5 min at 85°C. Gene expression was analyzed by RT-PCR using a 9800 Fast Thermal Cycler (Applied Biosystems). All 20×assay mixes were purchased from Applied Biosystems. Murine assays used were as follows: β2 microglobulin (Mm00437762_m1), surfactant A (Mm00499170_m1), surfactant B (Mm00455681_m1), surfactant C (Mm00488144_m1), surfactant D (Mm00486060_m1), CCSP (Mm00442046_m1), aquaporin-5 (Mm00437578_m1), albumin-1, (Mm00802090_m1) and ABCA1 (Mm00442646_m1). Rat assays included β2 microglobulin (Rn00560865_m1), surfactant B (Rn00684785_m1), surfactant C (Rn01466216_g1), albumin-1 (Rn00592480_m1) and ABCA1 (Rn00710172_m1). cDNA pre-amplification reactions was performed with the following reagents in a final volume of 50 µl: 12.5 µl of a pooled mixture for all assays (made by combining equal volumes all 20×TaqMan gene expression assays and diluting to a final concentration of 0.2×), 25 µl of TaqMan Preamp Master mix (Applied Biosystems) and 12.5 µl of cDNA. The reaction was performed on the 9800 Fast Thermal Cycler (Applied Biosystems) consisting of a 10-min cycle at 95°C followed by 14 cycles at 95°C for 15 s, then 60°C for 4 min. The final product was diluted with TE buffer (1 mM Tris [Invitrogen], 0.1 mM EDTA [Gibco] pH 8.0) and then kept at −20°C. All Real Time RT-PCR reactions were performed in 96-well plates on a 7900HT Fast RT PCR System with the following reagents in a final volume of 25 µl: 20×assay mix (for either β2 microglobulin or one of the target genes) and 2×TaqMan PCR Master Mix. Pre-determined amounts of cDNA were added to this mixture. Duplicate reactions of the target and housekeeping genes were performed simultaneously for each cDNA template analyzed. The PCR reaction consisted of an initial enzyme activation step at 95°C for 10 min, followed by 40 cycles of 95°C for 15 s and 60°C for 1 min. A cycle threshold value (CT) value was obtained for each sample, and duplicate sample values were averaged. The 2^−ΔΔCT^ method was used to calculate relative expression of each target gene (14). Briefly, mean CT value of target genes in each sample were normalized to its averaged housekeeping gene CT value to give a ΔCT value. This was then normalized to control samples (ΔΔCT), and the 2^−ΔΔCT^ value was obtained. To calculate 2^−ΔΔCT^ for target genes with no expression in the control group, a CT value of 40 was assigned to the control group so that a relative quantity of the target gene could be reported. The control group used for all comparisons was cells cultured for an equivalent period of time that had not been exposed to lung, liver or LDMV in culture.

### Immunohistochemistry

Five micrometer paraffin-embedded lung sections were deparaffinized, permeabilized with Proteinase K (Sigma) for 3 min at 37°C and fixed with 4% paraformaldehyde (Sigma) for 5 min at room temperature. Prehybridization buffer, which included dithiothreitol (Sigma) and CotI DNA (New England BioLabs) was applied to samples for 90 min at 37°C, then hybridization occurred using a proprietary digoxigenen-conjugated Y chromosome probe at 85°C for 10 min. Samples were then incubated at 45°C overnight. The following day, samples were labeled with an anti-digoxigenen Fluorescein isothiocyanate (FITC) antibody (Sigma, 1:33 dilution) for 60 min at room temperature, washed then labeled with rabbit anti-mouse prosurfactant C antibody (Abcam, 1:1,000 dilution) for 60 min at room temperature. Samples were then washed and labeled with an anti-rabbit Texas Red antibody for 15 min at room temperature, washed, counterstained with the nuclear label 4′-6-Diamidino-2-phenylindole (DAPI) and analyzed by fluorescence microscopy.

### Western blot

Protein was extracted from cells using the Pierce T-PER protein extraction reagent with Thermo Scientific Halt™ Protease Inhibitor single-use cocktail, per manufacturer's guidelines, and quantified using the Pierce BCA Protein Assay Kit, per manufacturer's guidelines, and the Nanodrop ND/1000 spectrophotometer. A total of 25 µg protein (per well), loaded onto Protein Pierce Precise™ Protein gels, were run at 150 V for 45 min at room temperature. Protein was transferred to Pierce Nitrocellulose Transfer Membranes using the Bio-Rad Mini Trans-Blot cell. Western blots for prosurfactant B protein (Abcam) were performed using the Pierce Fast Western blot Kit and Supersignal^®^ West Pico Substrate, per manufacturer's guidelines.

### Fluorescence microscopy

Samples were visualized using conventional and deconvolution fluorescence microscopy (Zeiss Axioplan 2 microscope; Carl Zeiss) at room temperature. Ten or more fields (63×magnification), which included 5,000–10,000 Y chromosome positive (Y+) cells, were analyzed for each lung section focusing on the presence or absence of prosurfactant C (Sp-C) co-signal. DAPI+cells that were also Y+ and Sp-C+, defined here as transplanted bone marrow-derived type II pneumocytes, were quantified. Selected sections were photographed at 40×or 63×magnification using the AxioVision software package (Carl Zeiss). Three-dimensional images were created of sample cells from a 25-layer (0.4 µm/layer) z stack to demonstrate co-localization of fluorescent signal. No photosubtraction or processing of the artifact was performed.

### Co-culture of LDMV with SILAC-labeled Lin– cells

Lin– cells were cultured either in SILAC^®^ RPMI-1640 labeling media, designated as “Heavy” media (containing 10% dialyzed FBS, supplemented with 100 mg/ml [U-13C6]-l-lysine (K+6), 100 mg/ml [U-13C6, 15N4]-l-arginine (R+10) and 100×l-glutamine (Invitrogen)) or in RPMI-1640 media (Invitrogen), designated as “Light” media (containing 10% dialyzed FBS, supplemented with 100 mg/ml l-lysine, 100 mg/ml I-arginine, 100×I-glutamine). Cells in the “Heavy” media were expanded for 6 doublings at 37°C to achieve complete labeling of cellular proteins with heavy labeled amino acids, while cells in “Light” media were independently maintained. LDMV were subsequently added to SILAC-labeled cells and this co-culture was maintained for an additional 7 days at 37°C. Cells grown in “Light” media were also maintained in culture for an additional 7 days but no LDMV were added. Cells were then collected and washed once with 1×PBS by centrifugation at 300*g* for 10 min.

### Protein extraction and mass spectrometry analysis of Lin– cells grown in “Heavy” and “Light” media

Cells were treated with CelLytic^®^ M reagent (Sigma) for 15 min. The lysed cells were centrifuged for 15 min at 16,000*g* to pellet the cellular debris and protein-containing supernatant was collected. The Micro BCA Protein Assay Kit (Pierce) was used to determine protein concentration. Protein samples from cells grown in “Heavy” and “Light” media were mixed 1:1 and run on a NuPAGE^®^ 4–12% Bis-Tris precast Gel (Invitrogen) by using XCell SureLock^®^ Mini-Cell (Invitrogen). The gel was stained with SimplyBlue^®^ SafeStain (Invitrogen) and the entire sample lane from the destained gel was cut into 10 equal gel pieces for fractionation. Each piece was washed 3 times with 50% acetonitrile/50% HPLC grade water prior to mass spectrometric analysis. Gel pieces were washed, reduced with DTT and alkylated with iodoacetamide. Gels were then digested with modified trypsin (Promega) overnight at 37°C with and the resulting peptide mixtures from each gel piece were analyzed separately by data dependent microcapillary reversed phase liquid chromatography tandem mass spectrometry (LC/MS/MS) LC/MS/MS was performed using an Easy-nLC nanoflow HPLC (Thermo Fisher Scientific) with a self-packed 75 µm id×15 cm C18 column coupled to a LTQ-Orbitrap XL mass spectrometer (Thermo Fisher Scientific) in the data dependent acquisition and positive ion mode at 300 nl/min. Generated MS/MS spectra were searched against the non-redundant Murine IPI database by using Mascot (Matrix Science). Protein quantitation was achieved by employing MSQuant software (http://msquant.alwaysdata.net/) with default settings.

### RNA extraction and microRNA microarray analysis of Lin– cells grown in “Heavy” and “Light” media

Total RNA was collected using the miRNeasy kit (Qiagen). Samples were sent to Dharmacon (Lafayette, CO, USA) microRNA microarray analysis. Analysis was performed on 150 ng of total RNA from each sample.

### Statistical analysis

Data were analyzed using the Student's t-test in cases where there were fewer than 6 measurements within 2 parent groups. Alternatively, Wilcoxon rank sum test was performed in cases where there were 6 or more measurements within 2 parent groups. We considered results to be statistically significant when p≤0.05. Data were presented as mean±standard error.

## Results

### Persistence of pulmonary epithelial cell gene expression, in vitro assay

**Fig. 1:**
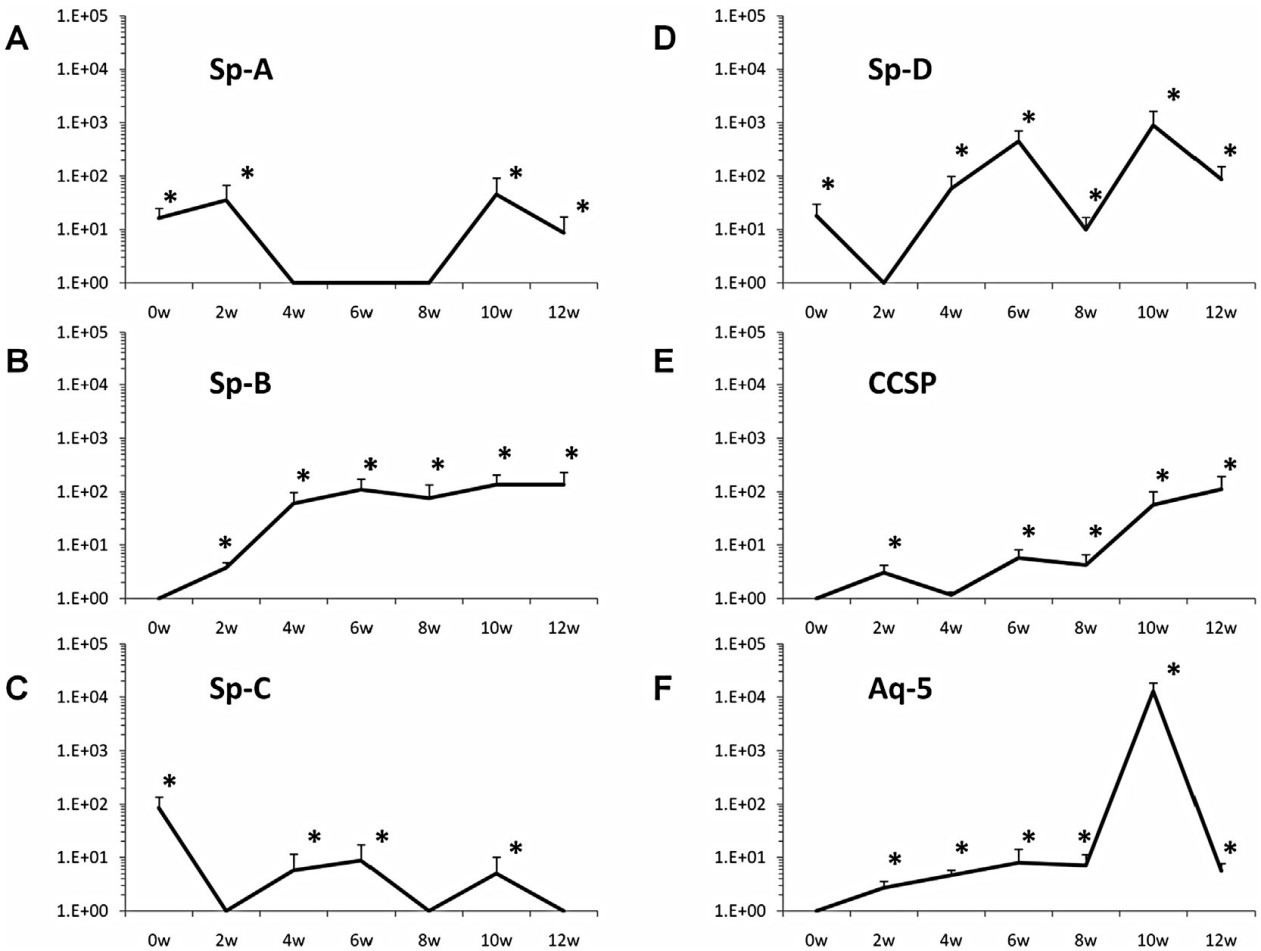
Pulmonary epithelial cell gene expression in marrow cells co-cultured with LDMV. Expression of (A) Sp-A, (B) Sp-B, (C) Sp-C, (D) Sp-D, (E) CCSP, (F) Aq-5 in WBM cells at various time points after an initial co-culture period of 1 week with LDMV. Data expressed as a fold difference compared with WBM cultured without LDMV for an equivalent period of time. *≤0.01, wilcoxon rank sum, 3 experiments, n=5–8, each time point.

**Fig. 2:**
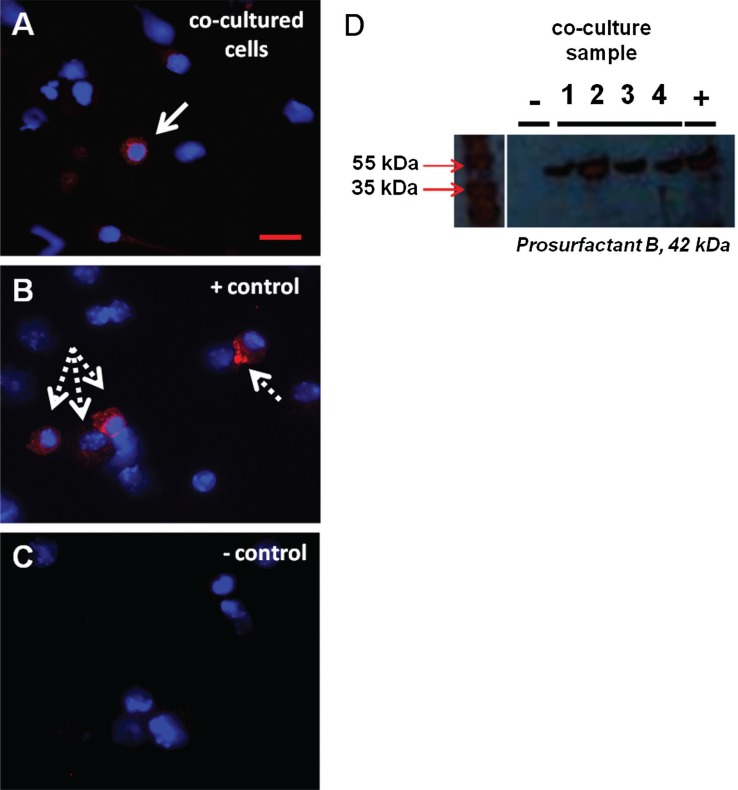
Pulmonary epithelial cell protein expression in marrow cells co-cultured with LDMV. (A) Prosurfactant B-expressing WBM cell (solid arrow) 12 weeks after exposure to LDMV in co-culture. (B) Prosurfactant B-expressing murine lung cell (dashed arrows). (C) WBM cells cultured without LDMV do not express prosurfactant B. (A–C) DAPI and Texas Red filters. (D) Western blot of WBM cells 12 weeks after exposure to LDMV in co-culture (lanes 1–4) and lung cells (+) express prosurfactant B whereas WBM cells cultured without LDMV (−) do not express prosurfactant B. Representative data from 1 of 3 experiments are shown.

WBM was co-cultured with murine lung fragments (separated by a 400 nm pore size cell-impermeable membrane), LDMV or cultured alone (control cells) for 7 days. Baseline pulmonary epithelial gene expression was determined (0 week time point) then WBM cells were washed and placed into secondary culture without lung or LDMV for 12 weeks. Pulmonary epithelial cell gene and protein expression was determined in WBM cells at 2-week intervals. Although expression varied, all pulmonary epithelial cell genes were detected and significantly elevated (vs. control cells) in cultured WBM cells exposed to lung (data not shown) and LDMV (Fig. 1A–F) 10–12 weeks after the initial co-culture period. In addition, prosurfactant B protein expression was detected WBM exposed to LDMV in co-culture up to 12 weeks after the initial co-culture period by immunohistochemical (Fig. 2A–C) and western blot (Fig. 2D) analysis. Altogether, these data indicate that LDMV-modified marrow cells are capable of persistent expression of pulmonary epithelial cell-specific genes and protein after several weeks in culture.

### Rat/mouse hybrid co-cultures

**Fig. 3:**
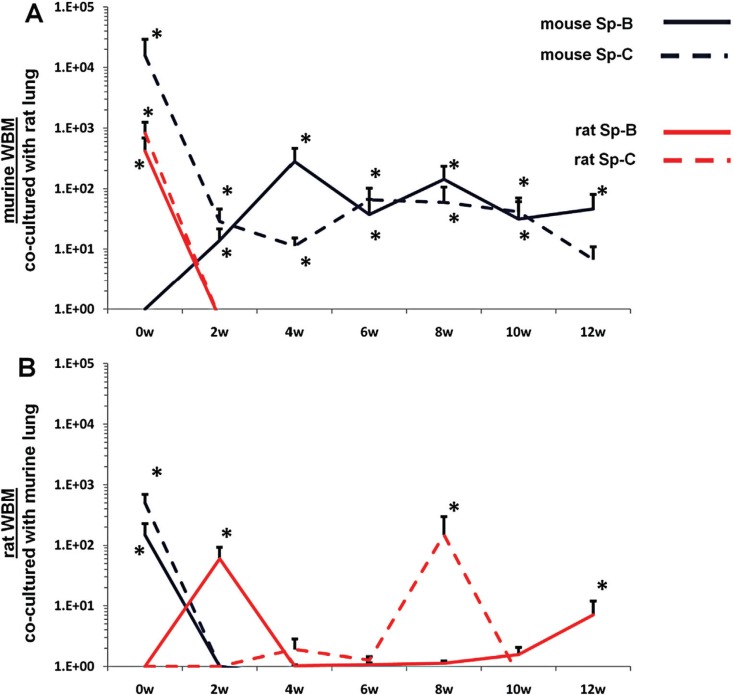
Rat/mouse hybrid co-culture, WBM and lung. Mouse-specific Sp-B (solid blue line) and Sp-C (dashed blue line), rat-specific Sp-B (sold red line) and Sp-C (dashed red line) expression in (A) murine WBM co-cultured with rat-derived lung and (B) rat-derived WBM co-cultured with murine lung. Data expressed as a fold difference compared with murine or rat-derived WBM cultured without lung for an equivalent period of time. *≤0.01, wilcoxon rank sum, 3 experiments, n=6–9, each time point.

**Fig. 4:**
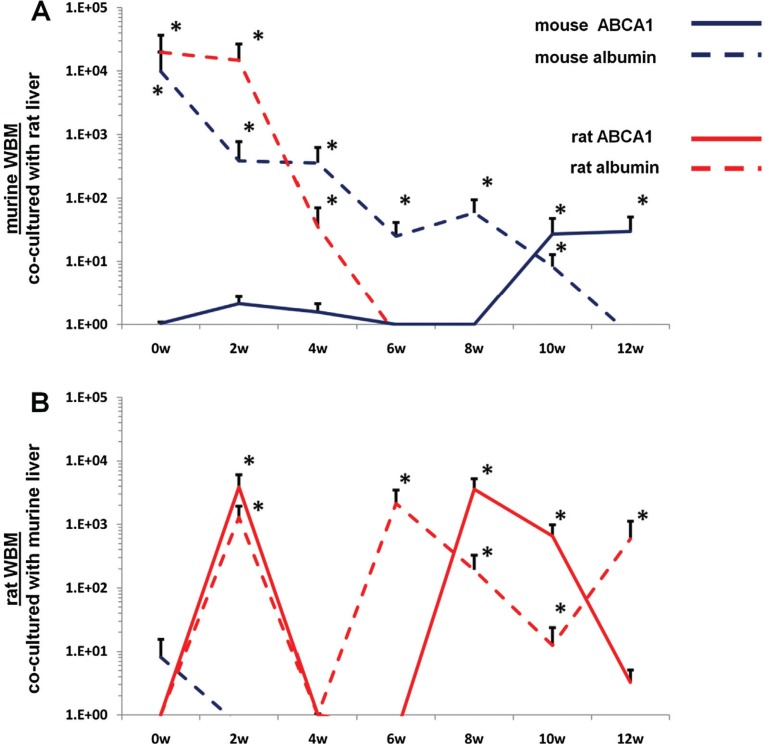
Rat/mouse hybrid co-culture, WBM and liver. Mouse-specific ABCA1 (solid blue line) and albumin (dashed blue line), rat-specific ABCA1 (sold red line) and albumin (dashed red line) expression in (A) murine WBM co-cultured with rat-derived liver and (B) rat-derived WBM co-cultured with murine liver. Data expressed as a fold difference compared with murine or rat-derived WBM cultured without liver for an equivalent period of time. *≤0.01, wilcoxon rank sum, 2 experiments, n=6, each time point.

WBM isolated from mice or rats were co-cultured with lung or liver fragments isolated from rats or mice or with no solid organs (control cells). Cells were separated by a cell-impermeable membrane and co-cultured for 7 days. Baseline gene expression was determined (0 week time point) then WBM cells were washed and placed into secondary culture without lung or liver for 12 weeks. Using species-specific (rat or mouse) primers, pulmonary epithelial cell and liver gene expression was determined in WBM cells at 2-week intervals by RT-PCR. In all co-cultures using murine WBM co-cultured with murine solid organs, only mouse-specific genes were detected. Likewise, in all co-cultures using rat WBM co-cultured with rat-derived solid organs, only rat-specific genes were detected, indicating that the PCR primers used were truly species-specific (data not shown). When murine WBM was co-cultured with rat-derived lung, surfactant that was expressed was both mouse and rat-specific at baseline (Fig. 3A). However, at later time points, up to week 12 of secondary culture, surfactant that was expressed was exclusively mouse-specific. Similarly, rat WBM co-cultured with murine lung expressed mouse and rat-specific surfactant at baseline but only rat-specific surfactant at later time points in secondary culture (Fig. 3B). When murine or rat-derived liver was substituted for lung in this co-culture system, albumin and ATP binding cassette transporter 1 (ABCA1) baseline expression in co-cultured WBM was specific to the species of animal from which the WBM and liver were isolated (Fig. 4A, B). At late time points in secondary culture, liver gene expression in co-cultured WBM was specific only to the species of animal from which the WBM was isolated. These data suggest that immediately after co-culture, the detection of mRNA species specific to the animal from which the co-cultured solid organ was derived represents the direct transfer of mRNA species to WBM cells in co-culture while detection of mRNA specific to the target marrow species is due to transfer of transcriptional activators. In later time points of secondary culture, detection of mRNA species specific only to the animal from which the co-cultured WBM was derived represents de novo synthesis of tissue-specific mRNA stimulated by transcriptional activators.

### Persistence of pulmonary epithelial cell gene expression, in vivo assay

Bone marrow cells, livers and spleens of lethally–irradiated female mice transplanted with WBM (isolated from male mice) co-cultured with LDMV or WBM cultured without LDMV were analyzed for the presence of pulmonary epithelial cell-specific gene expression 6 weeks after transplantation. Significant expression in these tissues was defined as a fold increase of 2.5 or greater compared to expression in marrow cells from mice transplanted with uncultured WBM (control). Significant expression of pulmonary epithelial-specific genes was detected in all tissues analyzed from mice transplanted with WBM co-cultured with LDMV (Table I). With the exception of 1 transplanted mouse, whose bone marrow expressed clara cell specific protein, no significant expression pulmonary epithelial cell-specific genes was detected in tissues from mice transplanted with WBM cultured without LDMV. These data demonstrate that LDMV-modified marrow cells are capable of persistent expression of pulmonary epithelial cell-specific genes in non-pulmonary tissue several weeks after transplantation.

**Table I:** In vivo persistence assay

		Percent (number) of transplanted mice with≥2.5 fold increase^a^ in expression of pulmonary epithelial cell genes					
		
Recipient tissue	Transplanted cells	Sp-A	Sp-B	Sp-C	Sp-D	CCSP	Aq-5
Bone marrow^b^	WBM+LDMV	41.2 (7/17)	5.9 (1/17)	0	35.3 (6/17)	11.8 (2/17)	0
	WBM cultured alone	0	0	0	0	8.3 (1/11)	0
Liver^c^	WBM+LDMV	25.0 (3/12)	0	0	0	50.0 (6/12)	16.7 (2/12)
	WBM cultured alone	0	0	0	0	0	0
Spleen^d^	WBM+LDMV	0	16.7 (2/12)	16.7 (2/12)	0	0	8.3 (1/12)
	WBM cultured alone	0	0	0	0	0	0

a Fold expression compared with WBM from control mice.

b Fold increase (range) of all pulmonary epithelial cell genes in bone marrow of transplanted mice: 2.54–58.9.

c Fold increase (range) of all pulmonary epithelial cell genes in livers of transplanted mice: 2.56–12.4.

d Fold increase (range) of all pulmonary epithelial cell genes in spleens of transplanted mice: 2.99–11.9.

**Fig. 5:**
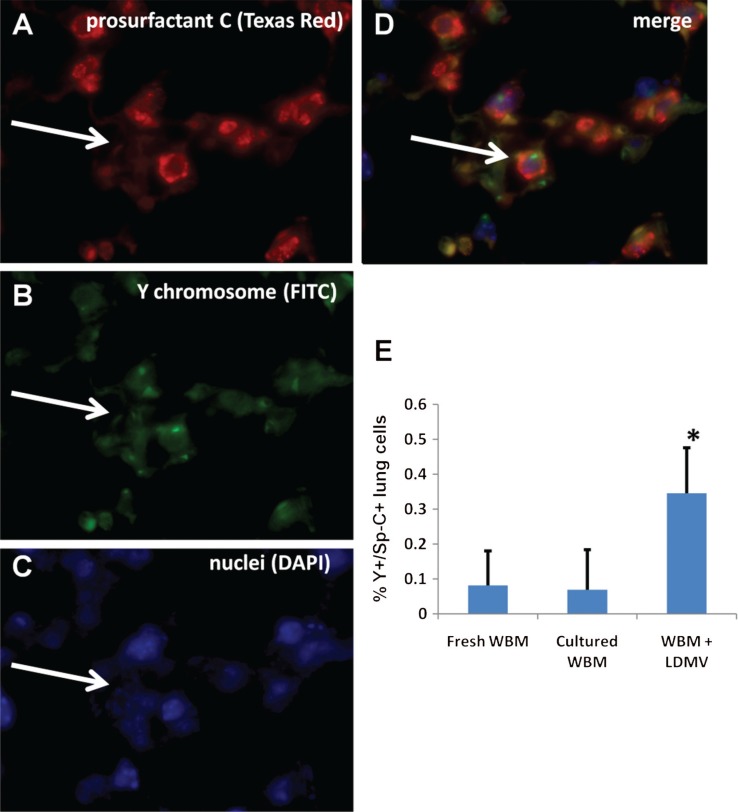
Transplanted marrow-derived type II pneumocytes. (A) Prosurfactant C+cell (white arrow, Texas Red filter), that is also (B) Y chromosome+(FITC filter) and (C) nucleated (DAPI filter) representing a (D) transplanted bone marrow cell-derived type II pneumocyte (merge) in the lungs of lethally irradiated female mice 6 weeks after transplantation with male murine WBM co-cultured with LDMV (63×magnification, room temperature). (E) Y+/prosurfactant C+cells, expressed as a percentage of all DAPI+lung cells, in mice transplanted with WBM co-cultured with LDMV, WBM cultured alone or uncultured WBM. *≤0.01, wilcoxon rank sum, 1 experiments, n=6–12.

Additionally, lungs from transplanted mice were analyzed for the presence of transplanted bone marrow cell-derived (Y chromosome+) type II pneumocytes (also prosurfactant C+, pro Sp-C+, Fig. 5A–D). There were over 5.5 times more Y+/pro Sp-C+cells in the lungs of mice transplanted with WBM co-cultured with LDMV compared with mice transplanted with WBM cultured alone (0.35±0.13% vs. 0.07±0.11% all DAPI+lung cells, p≤0.01, wilcoxon analysis, Fig. 5E). Mice transplanted with uncultured WBM had similar numbers of Y+/pro Sp-C+lung cells compared with mice transplanted with uncultured WBM (p≥0.05, wilcoxon analysis). These data demonstrate that LDMV-modified marrow cells preferentially contribute to the cellular component of the lung after transplantation into lethally–irradiated mice compared with unmanipulated marrow cells.

### Proteomic and microRNA profile of Lin– cells exposed to LDMV in co-culture

**Fig. 6:**
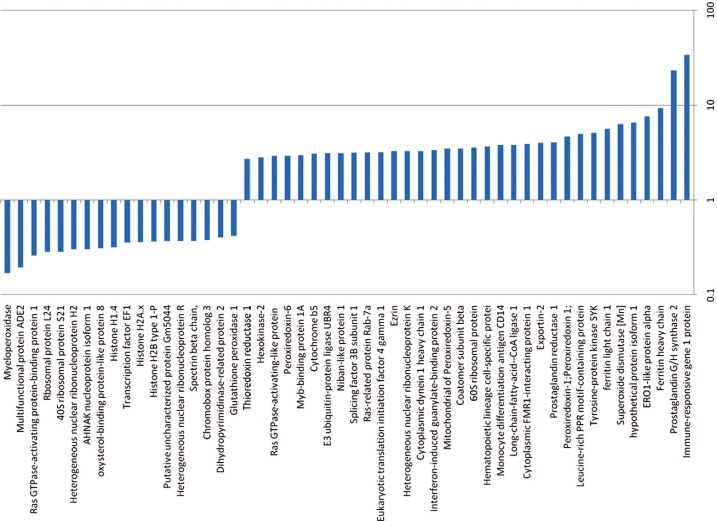
Altered proteome of Lin-cells co-cultured with LDMV. Relative expression of proteins in Lin– cells co-cultured with LDMV compared to Lin– cells cultured in the absence of LDMV. Up regulated proteins in LDMV co-cultured Lin– cells (34) are proteins common to both cell populations but more “Heavy”-labeled forms of these proteins were identified relative to “Light”-labeled forms. Down reregulated proteins (18) are proteins common to both cell populations but fewer “Heavy”-labeled forms of these proteins were identified relative to “Light”-labeled forms.

**Fig. 7:**
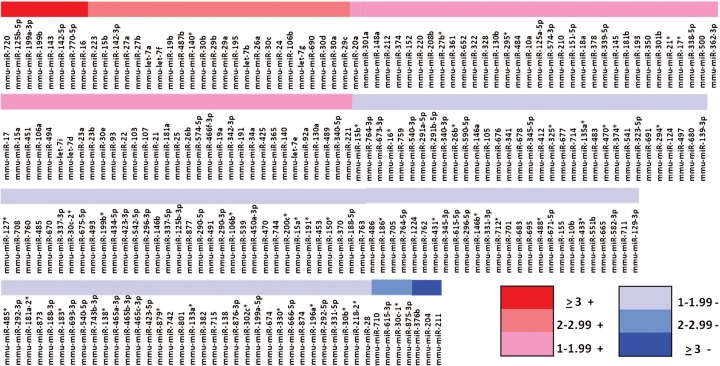
Altered microRNA profile of Lin-cells co-cultured with LDMV. Relative expression of microRNA in Lin– cells co-cultured with LDMV compared to Lin– cells cultured in the absence of LDMV. 159 species common to both cell populations are up regulated in LDMV co-cultured Lin– cells and 101 species common to both cell populations are down regulated in LDMV co-cultured Lin– cells.

Lin– cells co-cultured with LDMV for 7 days had a different proteomic profile compared with cells cultured in the absence of LDMV. We identified 34 proteins present in both cell populations but present in higher levels in LDMV-exposed cells as more of the “Heavy”-labeled forms of these proteins were identified relative to “Light”-labeled forms (p<0.01, Fig. 6). Conversely, we identified 18 proteins present in both cell populations but present in lower levels in LDMV-exposed cells as fewer “Heavy”-labeled forms of these proteins were found relative to “Light”-labeled forms. Additionally, we identified 159 microRNA species present in higher levels and 101 microRNA species present in lower levels in LDMV-exposed cells compared with cells cultured in the absence of LDMV (p<0.01, Fig. 7).

## Discussion

The stability of cellular phenotypes underlies much of the current understanding of cell biology. Stability is achieved on a global level in tissues due to their highly structured organization, with specific cell types carrying out specific functions. However, this does not mean that there is stability at the cellular level. Cells may be continually assuming different phenotypes to maintain tissue integrity, especially in the context of tissue injury. In prescient comments on the results of early hematopoietic stem cell research investigating the colony-forming unit spleen, Till, McCulloch and Siminovitch determined that there was total heterogeneity at the level of self-renewal from individual colonies, but relative stability when the system was considered globally (15). In discussing how this lax regulation (heterogeneity) could be reconciled with the orderly behavior of normal hematopoietic tissue, they drew an analogy with radioactive atoms: “If one studies a large number of radioactive atoms, one sees a very regular pattern of decay following an exponential law. However, if one studies individual atoms, they are found to decay in an unpredictable fashion at random. It appears possible that our studies of the progeny of single cells display the random feature of hematopoietic function, while study of large populations of cells reveals the orderly behavior of the whole system. From this point of view, it is the population as a whole that is regulated rather than individual cells”. This type of high cellular entropy and low organizational entropy may describe most of cell biology and microvesicle cell fate modulation provides a potential mechanism for such cellular flexibility.

Although earlier descriptions have questioned their biological relevance, microvesicles have since been viewed as possible immune modulators and mediators of thrombosis. More recent interest has focused on their capacity to shuttle cellular components from one cell to another and alter cellular fate. Transfer of CD41, integrins and CXCR4 between cells has been reported as has transfer of HIV and prions (16–20). Embryonic stem cell-derived microvesicles have been shown to reprogram hematopoietic progenitor cells by transfer of mRNA and protein (21). Apoptotic bodies from irradiated Epstein–Barr virus (EBV)-carrying cell lines were shown to transfer DNA to a variety of co-cultured cells resulting in integrated copies of EBV and subsequent expression of EBV genes in recipient cells (22). In addition, T lymphocyte extracts containing transcription factor complexes are capable of inducing fibroblasts to express lymphoid genes (23). Similarly, microvesicles from endothelial progenitors were shown to be capable of inducing a vascular phenotype in vitro and in vivo through delivery of microvesicles (12).

The biological relevance of microvesicles likely extends further as they may also play role in the pathogenesis of a variety of human diseases. In vitro culture studies done by our group and others have demonstrated that tumor-derived microvesicles can transfer determinants to non-malignant cells (16) and that human prostate cancer tissue is capable of inducing tissue specific mRNA transcription in human bone marrow cells (24). More recent work by Gatti et al. has shown that microvesicles derived from mesenchymal stem cells can mediate histological and functional recovery from acute renal tubular glycerol-induced injury or ischemia-reperfusion injury (25). Microvesicle entry into renal tubular epithelial cells appeared to facilitate these effects. Microvesicle-mediated transfer of RNA from human liver stem cells also stimulated liver regeneration in a sub-total hepatectomy model (26).

**Fig. 8:**
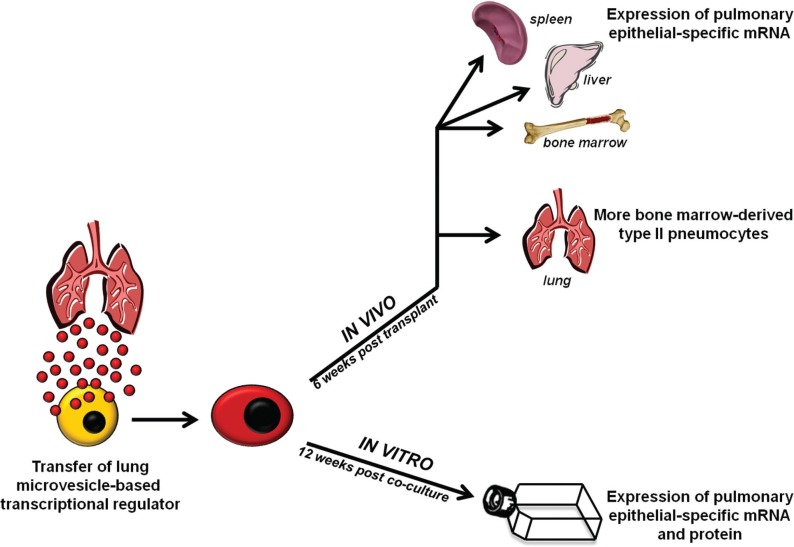
Summary of findings, in vitro and in vivo persistence assays. Lung cells shed microvesicle-based transcriptional regulators which are internalized by cells of the bone marrow. Changes in microvesicle-modified cells are persistent in vitro, as these cells continue to express pulmonary epithelial cell mRNA and protein up to 12 weeks later, and in vivo after transplantation into lethally-irradiated mice, as they lead to the expression of pulmonary epithelial cell mRNA in non-pulmonary tissue and preferentially engraft the lung as functioning type II pneumocytes.

The present work indicates that in the context of our culture system, microvesicle-induced changes in cellular phenotype are not transient, but rather a durable phenomenon as they persist over time in both in vitro and in vivo models (Fig. 8). Our data would suggest that the mechanism for these persistent changes is based on the transfer of a yet-to-be-determined transcriptional activator. Supporting this notion are data presented here showing that microvesicle-modified marrow cells have drastic changes in their microRNA profile and proteome. Transfer of a protein-based transcriptional activator with subsequent epigenetic change or transfer of microRNA or other non-coding RNAs which then act as a transcriptional modifier are among the likely possibilities. Regardless, the capacity of microvesicles to induce long-term phenotypic changes in cell populations is clear but the extent of these changes is also to be determined. We have demonstrated that transplantation of microvesicle-modified marrow cells into mice leads to over 5 times more marrow cell-derived type II pneumocytes compared with transplantation of unmanipulated marrow cells. In these studies, the absolute number of bone marrow-derived type II pneumocytes is low and represents a small proportion of all lung cells (0.35%) in these transplanted mice. Optimizing transplantation conditions (co-culture with a different number of microvesicles, different target cell population, lung injury model used, etc.) may lead to a greater degree of engraftment and this is an active area on interest with our group. These studies were not designed to assess the functional impact of these engrafted cells. Although it may seem unlikely that so few engrafted bone marrow-derived lung cells would improve lung function, others have demonstrated that lung engraftment does not always correlate with functional or histological change. Ortiz et al. demonstrated that transplanted mesenchymal stem cells attenuated histological distortion of the lungs of bleomycin-injured mice and did so with very little lung engraftment of transplanted cells (27). Local anti-inflammatory and immunomodulatory effects of these transplanted cells are believed to impart these beneficial changes and do so independent of significant lung engraftment. Additionally, we report that microvesicles derived from sources other than the lung (liver cells) have a similar impact on marrow cells, suggesting that these findings might represent a general biologic phenomenon. The genes selected for this analysis, albumin-1 and ABCA1, are highly expressed in liver tissue but not truly liver-specific (28, 29). Nonetheless, marrow cells exposed to liver-derived microvesicles have substantial upregulation of these genes, which is not seen in control cells. All together, the long-term persistence of microvesicle-induced phenotypic changes indicates that microvesicle-based cellular modulation might provide a powerful tool for tissue restoration strategies.
